# Is multimorbidity associated with risk of elder abuse? Findings from the AHSETS study

**DOI:** 10.1186/s12877-021-02347-y

**Published:** 2021-07-03

**Authors:** Jaya Singh Kshatri, Trilochan Bhoi, Shakti Ranjan Barik, Subrata Kumar Palo, Sanghamitra Pati

**Affiliations:** Department of Health Research, ICMR-Regional Medical Research Center, Bhubaneswar, Odisha 751023 India

**Keywords:** Multimorbidity, Elder abuse, Care dependence, Rural elderly, Geriatric care, LMIC

## Abstract

**Background:**

With an increasing number of older adults in low- and middle-income countries (LMIC), the burden of multimorbidity and functional dependence is on the rise. At the same time, a higher prevalence of elder abuse is observed in these populations. There is scarce evidence on the interplay between elder abuse and multimorbidity with no reports from LMIC settings yet. Present study examined the association of multimorbidity with the risk of elder abuse and its correlates in a rural elderly population of Odisha, India.

**Methods:**

The data for this study was collected as a part of our AHSETS study comprising of 725 older adults residing in rural Odisha, India. Multimorbidity was assessed by the MAQ PC tool while Hwalek-Sengstock elder abuse screening test (HS-EAST) was used to assess the risk of elder abuse. Functional dependence was measured by the Lawton IADL questionnaire. We used ordinal logistic regression models to identify the correlates of elder abuse and test for mediation by functional dependence.

**Results:**

Around 48.8 % (95 % CI:45.13–52.53 %) older adults had multimorbidity while 33.8 % (95 % CI:30.35–37.35 %) had some form of dependence. Out of 725, 56.6 % (CI 52.85–60.19 %) were found to be at low-risk elder abuse and 15.9 % (CI 13.27–18.72 %) being at high-risk. The prevalence of higher risk of elder abuse was greater among females, non-literates, widowed persons, those not currently working and those belonging to lower socio-economic strata. The risk of elder abuse was significantly associated with multimorbidity (AOR = 1.68; 95 %CI: 1.11–2.57) and functional dependence (AOR = 2.08; 95 %CI: 1.41–3.06). Additionally, we found a partial mediation mechanism of functional dependency between the pathway of multimorbidity and elder abuse.

**Conclusions:**

Elder abuse and multimorbidity are emerging as issues of significant concern among rural elderly in Odisha, India. Multimorbidity and functional dependence are associated with significantly higher odds of elder abuse among rural older adults. Further, we report the role of functional dependence as a partial mediator between multimorbidity and elder abuse. Therefore, potential interventions on reducing the economic, physical and care dependence among multimorbid patients may reduce the risk of elder abuse.

## Introduction

The global geriatric population is predicted to double from 11.5 % to 2015 to 22 % in 2050, with 80 % of them in the low and middle-income countries (LMICs), like India, where the majority live in rural communities [1, 2]. Older adults are at an increased risk of health challenges such as multimorbidity and functional limitations along with social concerns such as economic dependence, isolation and social security [3].

The prevalence of multimorbidity in this group is significant and ranges between 24–83 % in LMICs [4]. Elder abuse is also a growing, complex, and significant problem among these rural communities with multiple physical, social, cultural, economic and psychological dimensions. Elder abuse can be described as ‘‘a single or repeated act, or lack of appropriate action, occurring within any relationship where there is an expectation of trust which causes harm or distress to an older person” [5]. The prevalence of elder abuse varies across the globe which ranges from over 40 % in high-income countries to 13 %-28 % in LMICs [6]. Recent studies have reported the prevalence of elder abuse among rural populations to be as high as 50 % [7]. Elder abuse can manifest in many forms including physical assault, deprivation of food and healthcare, emotional abuse, involuntary confinement of isolation, financial abuse and legal abuse, etc [8]. While very few older adults seek help for abuse, there is insufficient evidence on the effectiveness of various interventions to address this issue in community settings [9–12]. There are many tools available for assessment of elder abuse with acceptable validity but none have been evaluated against measurable abuse and this heterogeneity leads to varying results [13, 14].

Multiple factors have been linked to an increased risk of elder abuse in rural communities such as older age groups, female gender, illiteracy, lower socio-economic status, marital status, and living arrangements [7, 15–17]. However, much of the evidence is from western countries and systematic reviews have shown that there is under representation of older adults from developing countries [18]. Robust prevalence studies in rural regions of LMICs are sparse making elder abuse a neglected priority in these countries [19]. In addition to this, multiple social, cultural and religious factors play crucial role in predisposing older adults to abuse and these factors vary significantly based on the socio-cultural milieu of the study site [20, 21]. Therefore we attempt to address this gap in a rural Indian setting.

There is considerable evidence suggesting multimorbidity is associated with functional dependence [22, 23]. On the other hand, dependence of the older adults, both functional and financial, is related to caregiver stress which in turn may contribute to an increased risk of elder abuse [24]. Therefore, a conceptual model with the above factors was hypothesized as a possible pathway for elder abuse. Against this backdrop, the present study was undertaken with an objective to examine the association of multimorbidity and functional dependency on the risks of elder abuse in a rural elderly population of Odisha, India.

## Methodology

### The AHSETS study

This study was carried out as part of the AHSETS study [22, 23]. The AHSETS study is a comprehensive effort to assess the health status of the rural elderly using a syndemic approach. The findings of the AHSETS study aim to inform policy interventions for the National Program of Healthcare of the Elderly (NPHE) in India such as routine screening practices for identifying, managing and preventing health and social disorders in rural communities [25].

### Study design, setting and population

We undertook a cross-sectional study, carried out in the rural block of Tigiria in Cuttack district, Odisha, India, between June 2019 and February 2020. Tigiria is an administrative block of Odisha, India, consisting of 52 revenue villages with a total population of 74,639 [26]. The study participants were residents of Tigiria block, aged more than or equal to 60 years who were conversant, comprehensible and provided their written informed consent to participate. We excluded seriously ill, bedridden patients as well as those with severe cognitive impairment. The details of the AHSETS study methodology is discussed elsewhere and we describe it briefly below [22, 23].

### Sample size and sampling

The minimum sample size required for the AHSETS study was calculated to be 725 [23, 27]. AHSETS study aimed to evaluate multiple health parameters among rural elderly and sample size was optimized to provide adequate power and precision for these estimates. For this hypothesis testing, based on our findings, for 95 % confidence levels the sample size provided a type-II error rate of 0.21 for a type-I error of 0.05. We used a cluster sampling technique to select 30 clusters (revenue villages) based on a Probability Proportional to Size (PPS), each cluster with a size of 25 sampling units. Systematic random sampling method was used in each of the clusters for identification of study participants.

### Data collection

Data were collected through interviews conducted by trained field investigators using a pre-tested tool and recorded on an Open Data Kit (ODK) form installed in android tablets. Multimorbidity was assessed using the Multimorbidity Assessment Questionnaire for primary care (MAQ-PC) tool developed and validated by Pati et. al. [28] This is an interviewer administered tool that identifies chronic diseases using a mix of physician diagnosed, self-reported and screening enquiry to categorize respondents with 2 or more chronic diseases as having multimorbidity. Elder abuse status was assessed using the pre-validated Hwalek-Sengstock Elder Abuse Screening Test tool. This included a series of questions to assess direct abuse, vulnerability and abusive situation administered in local language to the elderly and out of total score of 15, a score of more than equal to 3 was taken as the risk of being abused, neglected, or exploited. We further categorized the risk of abuse as No risk of abuse (≤ 2 score), Low risk of abuse (3–5 score) and high risk of abuse (≥ 6 score) [29]. Dependence for the activities was assessed using the validated Lawton IADL scale with a score range from 0 to 8. This tool categorizes elderly based on functional dependency as: dependent (score ≤ 2), partially dependent (score 3–6), and independent (score ≥ 7 for women or score ≥ 5 for men) [30]. Socio-demographic data were collected following standard census of India operational definitions. Socio-economic status (SES) was assessed using the per capita household income method outlined in the updated BG Prasad tool [31]. Economic Dependency was assessed as minimum monthly income as per the Government of India’s cut-offs from any source, including pension along with decision-making capacity to use this amount [32]. Familial structure of the participants was classified based on National family health survey definition into “single member”, “nuclear” and “joint” families [33].

### Quality control

Data collection was commenced after a comprehensive training of the study staff using a standardized manual of operating procedures (MOP) for the study and existing validated tools for the Indian population were used after their translation (and back translation) into the regional language, Odia, to ensure generalizability [34].

### Statistical analysis

Following data preparation and cleaning descriptive analysis was performed. The data was tested for normality. We used mean alongside the standard deviations to describe continuous variables and proportions with 95 % confidence levels for categorical variables. Bi-variate analysis was done using Chi-square test and Kendall-Tau ranked correlation.

We used regression models to test our hypothesis on the mediating role of functional dependency between multimorbidity and elder abuse. For this, we used the Judd & Kenny’s “Difference of Coefficients” approach to estimate the indirect effect of multimorbidity on elder abuse [35]. The assumption of proportional odds was tested using the Brant’s test and subsequently, the following 2 ordinal logistic regression models were built:

*Model-1:*

$$\mathrm{Logit}\;({\mathrm P}_{\mathrm{Risk}\;\mathrm{of}\;\mathrm{abuse}\;(0-2)})\;=\;{\mathrm\beta}_0\;+\;{\mathrm\beta}_1\;(\mathrm{Age}\;\mathrm{groups})\;+\;{\mathrm\beta}_2\;(\mathrm{Gender})\;+\;{\mathrm\beta}_3\;(\mathrm{Literacy})\;+\;{\mathrm\beta}_4\;(\mathrm{Dependence})\;+\;{\mathrm\beta}_5\;\\(\mathrm{Multimorbidity})\;+{\mathrm\beta}_6(\mathrm{Socio}-\mathrm{economic}\;\mathrm{status})\;+{\mathrm\beta}_7(\mathrm{Marital}\;\mathrm{Status})\;+{\mathrm\beta}_8(\mathrm{Type}\;\mathrm{of}\;\mathrm{Family})$$

*Model-2*

$$\mathrm{Logit}\;({\mathrm P}_{\mathrm{Risk}\;\mathrm{of}\;\mathrm{abuse}\;(0-2)})\;=\;{\mathrm\beta}_0\;+\;{\mathrm\beta}_1\;(\mathrm{Age}\;\mathrm{groups})\;+\;{\mathrm\beta}_2\;(\mathrm{Gender})\;+\;{\mathrm\beta}_3\;(\mathrm{Literacy})\;+\;{\mathrm\beta}_4\;(\mathrm{Multimorbidity})\;+{\mathrm\beta}_5(\mathrm{Socio}-\mathrm{economic}\;\mathrm{status})\\+{\mathrm\beta}_6(\mathrm{Marital}\;\mathrm{Status})\;+{\mathrm\beta}_7(\mathrm{Type}\;\mathrm{of}\;\mathrm{Family})$$

Where, P_Risk of Abuse_ = Probability of Elder Abuse (No risk, Low risk and High risk) & β_0_- β_8_ = Regression Coefficients. The indirect regression coefficient was calculated as given below, and its confidence intervals and significance levels were estimated using bootstrapping method.

$${\mathrm\beta}_{\mathrm{indirect}}\;=\;{\mathrm\beta}_4\;(\mathrm{from}\;\mathrm{model}-2)\;--\;{\mathrm\beta}_5\;(\mathrm{from}\;\mathrm{model}-1)$$

While we selected the primary exposure, mediator and outcome variables in the models based on our hypothesis (multimorbidity, dependence and elder abuse), variables that have been identified by previous reports to affect these primary variables of interest have been taken as confounders. Significant evidence is available separately on the socio-demographic covariates for multimorbidity [36–39], dependence [40–44] and elder abuse [18, 20, 45–47]. From among these covariates, we identified the following common variables that had significant associations (in either direction): Age, Gender, Socio-economic status, living status/marriage. Synonymous variables were added to the models above after testing them for collinearity. We tested the variables for correlation, tolerance, variance inflation and condition index but we did not find any collinearity among them. All analyses were performed using R statistical software packages (build ver. 4.0.3).

### Ethical considerations

Ethical approval was obtained from the institutional human ethics committee of ICMR-RMRC Bhubaneswar (Approval No- ICMR-RMRCB/IHEC-2019/022). Written informed consent was obtained from all participants and the national ethical guidelines for biomedical research were followed [34]. The data collected is stored electronically for a period of 5 years on secure institutional servers. Participant confidentiality was ensured during data collection. Vulnerable participants (such as those with dementia, unidentified diseases, and functional limitations) identified during the study were counselled and referred to the nearest primary care centers for further evaluation and management.

## Results

 We approached a total of 784 eligible households and with a non-response rate of 7.5 %, a total of 725 rural elderly people participated in the AHSETS study. Among them, around 48 % (*n* = 347) were female and the rest males. Age of the study participants ranged from 60 years to 106 years with a mean of 70.24 years (SD = 8.37). The prevalence of multimorbidity and dependence was found to be 48.8 % (95 % CI:45.13–52.53 %) and 33.8 % (95 % CI:30.35–37.36 %) respectively. The prevalence of low-risk abuse and high-risk abuse is 56.6 % (CI 52.85–60.19) and 15.9 % (CI 13.27–18.72) respectively.

We did not find any significant difference in risk of elder abuse among different age groups. However, high risk of elder abuse was seen more in females (22.2 %) as compared to males (10.1 %). The Demographic characteristics of study population are given below in Table 1.

**Table 1:** Demographic distribution of Elder abuse risk

Attributes		Elder Abuse N (%)			Total participants
		**High-Risk Abuse**	**Low-Risk Abuse**	**No Abuse**	
Age Group (Years)	> 90	1 (0.9 %)	8 (2.0 %)	3 (1.5 %)	12 (1.7 %)
	80–89	17 (14.8 %)	40 (9.8 %)	22 (11.0 %)	79 (10.9 %)
	70–79	35 (30.4 %)	92 (22.4 %)	44 (22.0 %)	171 (23.6 %)
	60–69	62 (53.9 %)	270 (65.9 %)	131 (65.5 %)	463 (63.9 %)
Gender	Male	38 (33.0 %)	202 (49.3 %)	138 (69.0 %)	378 (52.1 %)
	Female	77 (67.0 %)	208 (50.7 %)	62 (31.0 %)	347 (47.9 %)
Education	Illiterate	75 (65.2 %)	205 (50.0 %)	66 (33.0 %)	346 (47.7 %)
	Primary School	37 (32.2 %)	168 (41.0 %)	96 (48.0 %)	301 (41.5 %)
	Secondary School	1 (0.9 %)	19 (4.6 %)	18 (9.0 %)	38 (5.2 %)
	High School & Above	2 (1.7 %)	18 (4.4 %)	20 (10.0 %)	40 (5.5 %)
Occupation	Not Working	101 (87.8 %)	339 (82.7 %)	127 (63.5 %)	567 (78.2 %)
	Self Employed	5 (4.3 %)	22 (5.4 %)	27 (5.4 %)	54 (7.4 %)
	Agriculture	9 (7.8 %)	49 (12.0 %)	46 (23.0 %)	104 (14.3 %)
Marital Status	Currently Married	66 (57.4 %)	266 (64.9 %)	155 (77.5 %)	487 (67.2 %)
	Widowed	49(42.6 %)	137(33.4 %)	44(22.0 %)	230 (31.5 %)
	Never Married/divorced/Separated	0 (0.0 %)	7 (1.7 %)	1 (2.5 %)	8 (1.3 %)
Family Type	Single member	9 (7.8 %)	33 (8.0 %)	11 (5.5 %)	53 (7.3 %)
	Joint	50 (43.5 %)	246 (60.0 %)	119 (59.5 %)	415 (57.2 %)
	Nuclear	56 (48.7 %)	131 (32.0 %)	70 (35.0 %)	257 (35.4 %)
No. of Elderly In Household	1	57 (49.6 %)	209 (51.0 %)	95 (47.5 %)	361 (49.8 %)
	2 or more	58 (50.4 %)	201 (49.0 %)	105 (52.5 %)	364 (50.2 %)
SES	Low	79 (68.7 %)	223 (54.4 %)	87 (43.5 %)	389 (53.7 %)
	Lower-Middle	28 (24.3 %)	126 (30.7 %)	63 (31.5 %)	217 (29.9 %)
	Upper-Middle	7 (6.1 %)	52 (12.7 %)	35 (17.5 %)	94 (13.0 %)
	High	1 (0.9 %)	9 (2.2 %)	15 (7.5 %)	25 (3.4 %)
Economic Dependency	Dependent	95 (82.6 %)	323 (78.8 %)	117 (58.5 %)	535 (73.8 %)
	Independent	20 (17.4 %)	87 (21.2 %)	83 (41.5 %)	190 (26.2 %)
Chronic disease	No Chronic disease	11 (9.6 %)	84 (20.5 %)	37 (18.5 %)	132 (18.2 %)
	Single Chronic disease	36 (31.3 %)	152 (37.1 %)	51 (25.5 %)	239 (33.0 %)
	Multimorbidity	68 (59.1 %)	174 (42.4 %)	112 (56.0 %)	354 (48.8 %)
Functional dependency	Dependent	57 (49.6 %)	154 (37.6 %)	34 (17.0 %)	245 (33.8 %)
	Partially dependent	31 (27.0 %)	132 (32.2 %)	63 (31.5 %)	226 (31.2 %)
	Independent	27 (23.5 %)	124 (30.2 %)	103 (51.5 %)	254 (35.0 %)
Total		115 (15.9 %)	410 (56.6 %)	200 (27.6 %)	725 (100 %)

Similarly, bivariate analysis showed that those with multimorbidity (13.6 %) were at a significantly higher risk of elder abuse as compared to those with no chronic diseases (8.3 %), and functionally dependent older adults (23.3 %) were also at a higher risk of elder abuse as compared to those who were independent (10.6 %). The bivariate ranked Kendall-Tau correlation showed significant relationship between the dependency scores and elder abuse risk scores.

Among the domains of elder abuse assessed, responses predisposing to direct abuse were more frequent, except the response to a query on physical harm, which had the lowest frequency of all items. Most items to elicit potentially abusive situations had responses weighing negatively towards abuse. The frequency of responses for eliciting risk of elder abuse using the HS-EAST tool are listed in Table 2.

**Table 2:** Response directed to elder abuse

Item No.	HS-EAST Questionnaire items & Domains	Response for Elder abuse	
		Positive	Negative
**A. Direct abuse:**			
4	Who makes decisions about your life — like how you should live or where you should live?	404 (55.7)	321 (44.3)
9	Does someone in your family make you stay in bed or tell you you’re sick when you know you're not?	227 (31.3)	498 (68.7)
10	Has anyone forced you to do things you didn’t want to do?	145 (20.0)	580 (80.0)
11	Has anyone taken things that belong to you without your O.K?	200 (27.6)	525 (72.4)
15	Has anyone close to you tried to hurt you or harm you recently?	9 (1.2)	716 (98.8)
**B. Characteristics of vulnerability:**			
1	Do you have anyone who spends time with you taking you shopping or to the doctor?	73 (10.1)	652 (89.9)
3	Are you sad or lonely often?	359 (49.5)	366 (50.5)
6	Can you take your own medication and get around by yourself?	215 (29.7)	510 (70.3)
**C. Potentially abusive situation:**			
2	Are you helping to support someone?	301 (41.5)	424 (58.5)
5	Do you feel uncomfortable with anyone in your family?	53 (7.3)	672 (92.7)
7	Do you feel that nobody wants you around?	72 (9.9)	653 (90.1)
8	Does anyone in your family drink a lot?	102 (14.1)	623 (85.9)
12	Do you trust most of the people in your family?	65 (9.0)	660 (91.0)
13	Does anyone tell you that you give them too much trouble?	31 (4.3)	694 (95.7)
14	Do you have enough privacy at home?	285 (39.3)	440 (60.7)

Ordinal regression models were built for testing our hypothesis. We found that both multimorbidity and functional dependency increased the odds of elder abuse, in both unadjusted and adjusted analyses. This significant relationship of both the variables implies a partial mediation mechanism of functional dependency between the pathway of multimorbidity and elder abuse [48]. Details are provided in Table 3 below.

**Table 3:** Ordinal logistic regression model for odds of Elder abuse

Predictor	Unadjusted OR (95% CI)	Adjusted OR (95% CI)
**Chronic Diseases**		
Multimorbidity	**1.75**^**a**^ (1.27, 2.43)	**1.68**^**a**^ (1.11, 2.57)
Single Chronic disease	**1.58**^**a**^ (1.06, 2.38)	**1.85**^**a**^ (1.32, 2.58)
No Chronic disease	Ref	Ref
**Functional Dependency**		
Dependent	**2.28**^**a**^ (1.61, 3.22)	**2.08**^**a**^ (1.41, 3.06)
Partially dependent	**1.62**^**a**^ (1.14, 2.30)	1.38 (0.93, 2.06)
Independent	Ref	Ref

Variables added for adjusted ORs are: Gender, Age group, Literacy, Type of family, Marital status, Socio-economic status

^a^Significant at alpha =0.01 level

## Discussion

This study was carried out among 725 rural elderly people and we found a significant proportion of the study population were having multimorbidity, functional dependency and were at risk of elderly abuse. The prevalence of low and high risk of elder abuse was found to be around 56.6 and 15.9 % respectively. Females (82.1 %) were more at risk of abuse than males (63.5 %). Increased risk of elder abuse was found to be associated with presence of chronic diseases (and multimorbidity), functional dependency, staying without a family, lower socio-economic status, and economic dependence.

While there have been studies assessing elder abuse in isolation, we evaluate its relationship with multimorbidity through the mediating pathway of functional dependence. Reviews have reported a pooled prevalence of elder abuse of 15.7 % (95 % CI 12.8 to 19.3) and this is generally higher in developed countries (44.6 %) as compared to LMICs (13.5–28.8 %) [6, 19]. A review specific to Asia reported highest prevalence in China (36.2 %) and lowest in India (14 %) [20]. Prevalence of elder abuse was highly varying across different countries such as Nepal, Singapore, Brazil, Iran, and Malaysia ranging between a high of 61.7 % to a low of 4.5 % [19, 20, 49–53].

Similar heterogeneous results have been reported from India as well. While rural community-based studies from south India in Kerala and Puducherry have found a prevalence of elder abuse to be high at around 60 and 50 % respectively, other parts of India reported a prevalence between 9.31 and 35 % and studies in old age homes in Karnataka and Chennai have found a prevalence of 35.2 and 21.75 % [7, 16, 17, 54–59]. This is probably because, unlike assessment of multimorbidity, which tends to be relatively precise, the prevalence of elder abuse varies significantly with the tool used to assess the same.

We have used the HS-EAST tool to assess elder abuse which is the first such tool to be developed and validated. It is unique in that it gives a quick classification based on the presumed risk of abuse and subsequently more useful in targeting further evaluation and preventive measures [13].

Similar to our findings, few other studies that aimed to assess burden of elder abuse have also reported that multimorbidity and functional dependency significantly increases the risk of elder abuse [60–64]. The pathways via which multimorbidity increases the risk of elder abuse, as evident in our study, may be related to the fact that multimorbidity leads to increased care dependence, healthcare utilization, frailty and functional dependence [22, 65]. Therefore, potential interventions on reducing the economic, physical and care dependence among multimorbid patients may have potential to additionally reduce the risk of elder abuse.

People with cognitive impairment were excluded and this is a potential limitation as they are likely to be at higher risk for elder abuse. The study is also limited by its study population being rural elderly and any generalizations to urban residents should be done cautiously. Follow-up qualitative assessments are necessary to understand the mechanisms and outcomes of those at risk of elder abuse.

The prevention, diagnosis, and control of multimorbidity and elder abuse need to be addressed at family and community level through existing primary health care system. Our findings suggest that an integrated approach to identify and manage these complex issues of older adults would give better returns. While the National Program for Health care of the Elderly in India adopts a strategy for community-based screening for chronic conditions, addition of functional ability and elder abuse into the program will be useful. With the current National Policy on Older People in India not looking into the issues faced by care providers, additional provisions in the program for caregiver counselling and support may be helpful to address the issues faced by them in case of dependent older adults. Implementation research to test the feasibility and effectiveness of using comprehensive screening tools that include multimorbidity, dependence and elder abuse appears to be necessary.

## Data Availability

The datasets used and/or analyzed during the current study are available from the corresponding author on reasonable request.
